# Assessing the Application of Physiologically Based Pharmacokinetic Models in Acute Chemical Incidents

**DOI:** 10.3390/jox15020042

**Published:** 2025-03-11

**Authors:** Sydney Boone, Wenjie Sun, Pavani Gonnabathula, Jennifer Wu, Maureen F. Orr, M. Moiz Mumtaz, Patricia Ruiz

**Affiliations:** 1 Oak Ridge Institute for Science and Education (ORISE), Oak Ridge Associated Universities (ORAU), Oak Ridge, TN 37830, USA; 2 Office of Innovation and Analytics, Simulation Science Section, Agency for Toxic Substances and Disease Registry (ATSDR), Centers for Disease Control and Prevention (CDC), Atlanta, GA 30329, USA; 3 Office of Innovation and Analytics, Registries and Surveillance Section, Agency for Toxic Substances and Disease Registry (ATSDR), Centers for Disease Control and Prevention (CDC), Atlanta, GA 30329, USA; 4 Office of Associate Director for Science, Agency for Toxic Substances and Disease Registry (ATSDR), Centers for Disease Control and Prevention (CDC), Atlanta, GA 30329, USA

**Keywords:** physiologically based pharmacokinetics, PBPK, toxicokinetics, acute chemical incidents, vinyl chloride, chemical exposures, surveillance, chemical releases, computational modeling, acute exposure

## Abstract

Chemical release incidents in the United States involve hazardous chemicals that can harm nearby communities. A historical tracking of these chemical release incidents from 1991 to 2014 across up to 16 states has been conducted by The Agency for Toxic Substances and Disease Registry (ATSDR), utilizing the Hazardous Substances Emergency Events Surveillance (HSEES) and the National Toxic Substance Incidents Program (NTSIP) systems. By analyzing surveillance data, patterns of these different chemical releases can be identified to develop and construct a health-protective course of action. Physiologically Based Pharmacokinetic (PBPK) models can simulate chemical exposures during acute chemical incidents. For a retrospective study of an acute chemical release in 2012, we examined the components necessary to integrate PBPK-modeled exposure assessments in ATSDR’s Assessment of Chemical Exposure (ACE) program. We focused on data from a published investigation of vinyl chloride (VC) exposure to assess the utility of PBPK in evaluating exposures among residential populations near the release site. The initial estimate from the real-time air monitoring at the release site revealed that air levels greatly exceeded the Acute Exposure Guideline Levels (AEGL) of 1200 ppm, with PBPK models predicting corresponding VC blood levels of 3.17 mg/L. “Real-time” and “after-action” air modeling estimated VC levels at various distances from the release site over time. PBPK modeling provided insight into possible residential blood levels of VC over several days following the incident. These findings indicate that PBPK modeling could be valuable for reconstructing exposure scenarios associated with acute chemical releases.

## 1. Introduction

Environmental chemical incidents involve the uncontrolled release of harmful substances into the environment, posing potentially widespread risks to ecosystems and human health [1,2,3,4,5,6,7,8]. These incidents can occur from various sources, such as industrial accidents (including leaks, spills, and explosions), transportation accidents (involving trucks, trains, or ships carrying hazardous materials), natural disasters (which can damage storage facilities or disrupt containment measures), illegal dumping, and agricultural practices that can contaminate soil and water [1,4,8,9,10,11]. The fate and transport of harmful chemicals released depend on various transformation and degradation processes such as chemical and biological degradation, volatilization, leaching, adsorption, and movement mechanisms in air, water, and soil, which is crucial for assessing potential risks to human health and ecosystems [4,7,8,12]. The effects of these chemical incidents may be immediate and acute or chronic health issues that develop over time. The severity of these effects depends on various factors, including the chemical dosage, exposure duration, and routes of exposure, such as dermal, ingestion, and inhalation [1,4,6,8,9,10,13]. There is a growing concern regarding the potential health implications of accidental chemical releases, particularly for vulnerable populations such as children, pregnant women, the elderly, and those with chronic health conditions who may be more sensitive to chemical exposure.

The Agency for Toxic Substances and Disease Registry (ATSDR) is mandated to investigate the impact of chemical exposures using state-of-the-art applied toxicology [14]. Often this includes simulation science tools [15,16]. For years, ATSDR has gathered data on these incidents, including factors contributing to the release, the properties of chemicals, the routes and amount of chemicals released, the number of people injured, health effects experienced, response actions, and potential community exposure [5,9,10,17,18]. In 2010, ATSDR launched a surveillance data system called the National Toxic Substance Incidents Program (NTSIP) [19], which included nine states—California, Louisiana, North Carolina, New York, Missouri, Oregon, Tennessee, Utah, and Wisconsin—in a four-year program. Since participation in data collection was voluntary, the findings do not fully represent the scope of chemical incidents across the United States [1,4,5,9,19].

NTSIP defined an acute incident as an uncontrolled or illegal acute release of any toxic substances that poses a health risk. Each participating state recorded data about these events in an online portal, where basic information on the event were maintained. NTSIP staff consulted national, state, and local agencies for additional data sources and utilized geographic information systems to identify vulnerable areas near these incidents [1,19].

Basic information was classified as follows:

- Type of toxic substances: chemical, biological, radiological, or medical;
- Categories of affected population (age, employment);
- Location of event (classified according to the North American Industry Classification System [NAICS]);
- Level of personal protective equipment used—four levels ranging from most to least protective;
- Routes of chemical release: volatilization, spill, fire, or explosion;
- Season of year: spring, summer, fall, or winter;
- Time and duration of the incident: either from 6:00 pm to 5:59 am or from 6:00 am to 5:59 pm.

### Computational Tools for Environmental Chemical Incidents and Chemical Risk Assessment

Chemical releases into the environment pose serious risks to human health and ecosystems. Fortunately, advancements in computational tools and a better understanding of molecular and cellular toxicity mechanisms have been crucial in addressing these challenges. These developments have led to the creation of innovative computational methodologies, including:

- Fate and transport modeling and simulation: atmospheric dispersion, hydrodynamic, and fate and transport models help predict how chemicals move through air, water, and soil, facilitating informed decision-making.
- Data analysis and visualization: Geographic Information Systems (GIS), remote sensing technologies, and environmental databases offer essential data visualization and analysis, enhancing situational awareness and emergency response planning.
- Decision Support Systems (DSS): real-time monitoring systems, early warning systems, and risk assessment tools like ALOHA and RMP simulate chemical release scenarios, aiding in rapid response and mitigation strategies.
- Physiologically based pharmacokinetic (PBPK) modeling: PBPK models are helpful in assessing and linking chemical exposure to the human body. In the context of chemical incidents, these models are employed to predict the body burden of chemical exposures.

PBPK modeling can be used to estimate the internal dose of a chemical in the body after a chemical exposure by humans or animals, focusing on specific target organs or tissues [3,20]. PBPK models are valuable tools in risk assessment because they help predict how chemicals behave in the body, which is crucial for evaluating potential health risks. PBPK are valuable tools that can be used to simulate the absorption, distribution, metabolism, and excretion (ADME) of chemicals across various tissues and organs, and the impact of various physiological and genetic factors on chemical behavior [21,22,23,24]. PBPK models could support exposure investigations and generate data that enrich the research database [2,3,14,16,25,26,27]. This information can inform future studies evaluating the impact of chemical incidents.

In addition to PBPK models, several open-source computational tools (see Supplementary Table S1) are available for mapping emergency chemical incidents, and identifying affected areas and locations that can be utilized in future studies. These software programs can enhance research efficiency, potentially reducing the number of people affected by chemical incidents and allowing for more comprehensive studies.

This study investigates the application of PBPK modeling in case studies related to chemical incidents within the NTSIP framework. The goal is to determine whether PBPK models can be effective tools for understanding and predicting the behavior of hazardous chemicals in the human body during acute exposure scenarios, thus contributing to more informed and robust chemical risk assessments.

## 2. Methods

### 2.1. NTSIP Data

NTSIP collected data on incidents involving acute toxic substances, including injuries and fatalities resulting from exposures [5,19]. These data encompassed the transportation model linked to each chemical incident, the phase of transportation, the types of injuries, the number of chemicals involved, the time of day, day of the week, and season. They also identified the primary contributing factor and the locations of at-risk populations [5,19]. We compiled and analyzed NTSIP data to produce clear tables detailing acute toxic substance incidents and associated casualties.

Additionally, we provided information on the ten most frequently involved individual chemicals by year, categorized by transportation and fixed facilities. We examined incidents and affected individuals according to primary and secondary contributing factors, the number of people involved in each incident, and the types of incidents. NTSIP data were used to select one of the most common chemicals for a case study.

#### 2.1.1. Case Study

We conducted a retrospective analysis of an acute chemical release resulting from a train derailment to explore the components necessary for integrating PBPK-modeled exposure assessments in ATSDR’s Assessment of Chemical Exposure (ACE) program [4,12] (https://atsdr.cdc.gov/ace/php/about/index.html, accessed on 3 December 2024). A punctured tanker car discharged approximately 24,000 gallons of vinyl chloride (VC) near a small town. A shelter-in-place order was issued and subsequently lifted and reestablished multiple times over four days, as VC levels in the air varied with weather conditions. VC is a colorless gas with a faint, sweet smell. Its main uses are various vinyl goods and polyvinyl chloride (PVC) plastic. However, inhaling vinyl chloride can have serious negative health effects, including the development of various cancers such as liver angiosarcoma, hepatocellular carcinoma and cholangiocellular carcinoma. It can also cause hepatic issues like fibrosis, cirrhosis, and steatohepatitis. Additionally, exposure may result in neurological effects such as peripheral neuropathy, as well as immunological impacts characterized by increased levels of circulating immune complexes, immunoglobulins, complement factors, and inflammatory cytokines, among other concerns [28]. Collaborating with the state and local health departments, the ACE team surveyed community members who may have been exposed, gathered information from hospital staff treating VC exposed patients, surveyed personnel from a facility cut off by the derailed train, and conducted hospital chart reviews. State partners also distributed a survey to all households in the community.

A train derailed, resulting in VC puncturing, was most likely released as a liquid, aerosol (mist), and gas (vapor). Measurements were taken shortly after the derailment through air monitoring. The first measurements near the scene were as high as about 1400 ppm. Over the next couple of hours, vinyl chloride concentrations fluctuated near the scene, ranging from tens to hundreds of ppm, and decreased and increased over the next two days. Also, spatiotemporal prediction models that use statistical and machine learning techniques to predict air pollution levels at specific locations and times, such as air dispersion models, provide estimates of the range of vinyl chloride concentrations that could have been in the air in the first hour after the vinyl chloride leak began. The National Oceanographic and Atmospheric Administration (NOAA) and EPA developed the Areal Locations of Hazardous Atmospheres (ALOHA) model as a tool for emergency responders and planners to estimate how chemicals may be dispersed in the air following a release [29]. As part of the response to the train derailment in Paulsboro, the NOAA Office of Response and Restoration developed a series of ALOHA models to assist emergency responders in evaluating the threat posed by the vinyl chloride release. Models developed for the Paulsboro train derailment indicated that much of the vinyl chloride would have escaped the tank within the first hour [30,31]. The models also estimated that outdoor air concentrations within the first hour could have exceeded 4800 ppm as far out as 0.2 miles in the direction of the wind, 1200 ppm as far out as 0.4 miles, and 250 ppm as far out as 0.8 miles [30]. Much of the area of Paulsboro is within 0.8 miles of the train derailment [32].

Estimating with confidence the VC concentrations to which people were exposed is complicated by temporal and geographic gaps in available monitoring and air dispersion modeling data, differences in methods employed to measure VC levels, differences in averaging times of measurements, and the variability introduced by topography and wind direction. Many residents living further from the scene could have been exposed to VC concentrations as high as 250 ppm in the first hours. Adults and children were exposed to varying levels of vinyl chloride [32].

As a result of this release, 250 hospital visits occurred. Of these visits, 237 patients were examined by a physician, 231 (97.5%) were treated in the emergency department, and 6 (2.5%) were admitted. Five admitted patients had preexisting medical conditions. Thirteen of 14 asymptomatic patients were children under the age of 10. One hundred forty-five patients (62.8%) discharged were diagnosed solely with exposure to vinyl chloride [33].

#### 2.1.2. Train Derailment

**Figure 1:**
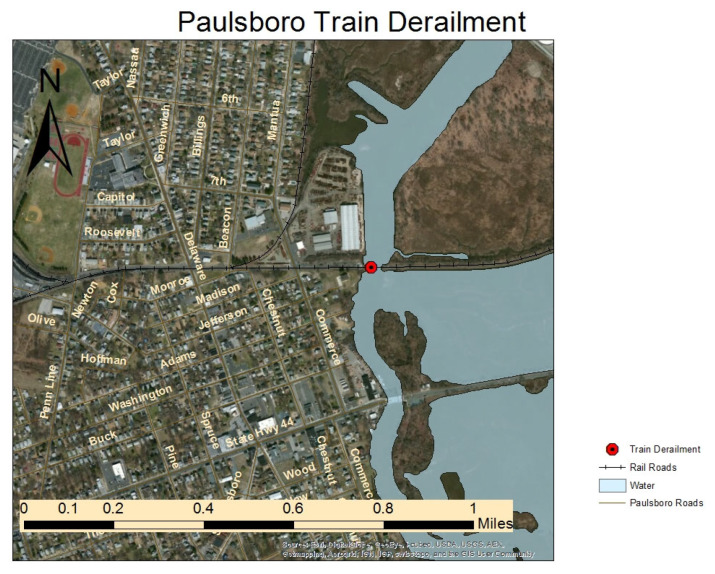
The site map of the train derailment for vinyl chloride derailment, which reported by the Division of Epidemiology, Environmental and Occupational Health New Jersey Department of Health in their 2014 Health Consultation. (https://www.state.nj.us/health/ceohs/documents/eohap/haz_sites/gloucester/train_derail/air_quality_report.pdf accessed on 3 December 2024) [29].

We utilized the Paulsboro, New Jersey health consultation [32], carefully analyzing all tables and figures, including those detailing the symptoms reported and the individuals who experienced them (Figure 1). In addition, we thoroughly examined tables outlining the locations where volatile organic compounds (VOC) concentrations were measured and the techniques used for their collection (Table S2).

The health consultation indicated that the VOC concentration was recorded on the day of the derailment and six days later. We applied the conversion factor to calculate the corresponding VC concentrations (Tables S2–S4).

### 2.2. ATSDR PBPK Modeling Tool

Briefly, ATSDR utilized Berkeley Madonna (BM) 10.4.2 software to develop a human PBPK toolkit, a library of models for several priority pollutants, including volatile organic chemicals (VOCs) [25,34,35]. This toolkit is used for screening purposes, particularly in chemical incident scenarios, when no other tools are available. Several criteria were used in the selection of the models for the toolkit, including model availability, performance, accuracy, reproducibility, the number of data sets used for calibration and evaluation, the model maturity (the number of predecessor models from which it was derived), and the author’s experience. We derived a generic model that could be used for several VOCs, including VC, by substituting chemical-specific parameters. The PBPK VC model was recoded from the original PBPK model published by Clewell et al., which met the above-mentioned study criteria. The Clewell et al. model [32] has been used and accepted by the USEPA and ATSDR for developing health guidance values for VC and met the above selection criteria. This model used well-mixed flow-limited compartments describing the mass balance of the chemicals in multiple tissues. We recoded this model into a generic model. This VC-recoded PBPK model enables the simulation of various exposure routes, individually or simultaneously.

Only parent compound data sets and accompanying simulations were extracted from figures reported in the original model and used to evaluate the generic model simulations. Thus, the original model simulations for metabolites and metabolite data were not included in the generic PBPK model for VOC concentration predictions. The performance of the VC-recoded model was found adequate based on a comparison with the published human kinetic data for VC. To further evaluate the reliability of our generic VC model, we also calculated the area under the concentration curve (AUC).

The VC air concentrations obtained from the exposure investigation conducted in Paulsboro, New Jersey, were then used as input exposure doses in the VC PBPK model to predict the arterial blood concentrations in the exposed population at the following intervals:

- A few hours after the derailment (Table S2);
- The subsequent 24 h post-derailment (Tables S3 and S4).

Simulations related to the Paulsboro spill primarily focused on the inhalation exposure pathway, as the majority of exposure occurred via inhalation [36]. Additionally, we used the VC PBPK model to simulate exposure at Acute Exposure Guideline Level (AEGLs) (high exposure dose) based on the exposure investigation conducted in Paulsboro, New Jersey (Table S5).

## 3. Results

### 3.1. NTSIP and Train Derailment Data

The NTSIP data were organized in multiple ways to highlight the types of chemicals with the greatest impact and to identify the most frequent chemical incidents. The chemical category most frequently associated with chemical spills was the VOCs class (Table 1 and Table 2). We analyzed the Paulsboro health consultation train derailment data to obtain VC levels as input exposure doses for the PBPK model.

The number of incidents and their corresponding frequencies were recorded in Table S3. Notably, 2014 (the final year of data collection) recorded the lowest number of incidents. The total number of chemicals involved in each incident was also provided, with most incidents involving only one chemical, while very few resulted from more than two chemicals. Additionally, Table S3 presents the frequencies of different types of releases, including the total number of events, events with injuries, the number of injuries and fatalities, categorized by event type: fixed facility (F) or transportation-related (T).

### 3.2. ATSDR PBPK Model Tool

**Figure 2:**
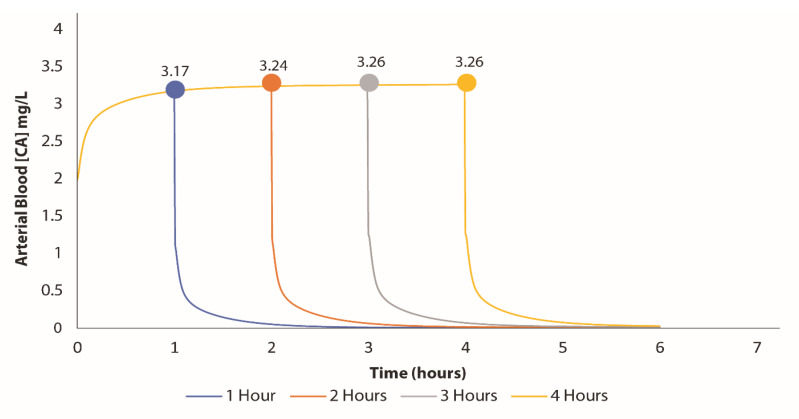
Predicted arterial blood concentrations after 4 h of exposure following the derailment. Samples of vinyl chloride were collected after the first hours of the train derailment. The samples were converted (using the conversion factor of VC) and used in Berkeley Madonna to generate the predicted arterial blood concentration (at the unit of mg/L).

Our BM VC PBPK model was used to simulate four hours of exposure to determine the peak VC concentration in the blood for each hour following the initial incident. According to the exposure investigation, the AEGL-2 concentration for a 60 min exposure was approximately 1200 ppm, which was applied in one of the parameter settings. The model estimated the arterial blood concentration to be around 3.17 mg/L after an hour of exposure. As illustrated in Figure 2, there were slight increases in concentration over time as exposure continued.

The results from the air samples were utilized to estimate the concentration of arterial blood in individuals exposed on the day of the derailment and in the following days, both outside and inside the evacuation area. The peak VC concentration (57 ppm) occurred on December 3, leading to the highest arterial blood (CA) concentration of 0.142 mg/L for outside exposure. For inside exposure, the highest concentration of VC observed was 1649.2 ppm on December 4, which also corresponded to the highest arterial blood (CA) concentration of 4.7 mg/L. The relationship between VC concentration and arterial blood concentrations is presented in Table 3.

PBPK models can also facilitate reverse dosimetry by estimating the external exposure dose in reverse based on available biomonitoring data, such as chemical levels measured in blood or urine samples from an exposed population [34].

AEGLs are used to help protect the public when there has been a chemical release that is short-term in duration. AEGLs estimate how the general public would react to a release of this nature, so they can be used to identify areas where a hazard exists if the substance hazardous concentration is exceeded for the specified exposure duration. For example, in areas with concentrations just above the AEGL-1, most people would experience temporary, non-disabling effects. On the other hand, in areas with concentrations just above the AEGL-2, most people would experience significant (but not life-threatening) health effects. In AEGL 3, people could experience life-threatening health effects or death. Typically, the AEGL values will be different for each exposure. This is because the physical effects are typically related to dose (that is, concentration over exposure duration). However, in some cases, the AEGL values will be the same for some durations. This situation usually occurs, as in VC at the AEGL-2 and 3 levels (as in VC AEGL data), because it is a threshold for disabling and death effects; some effects depend only on concentration, not on the length of time people are exposed.

Several trends can be observed in VC AEGLs (https://www.epa.gov/aegl/vinyl-chloride-results-aegl-programs, accessed on 3 December 2024). First, as one moves from AEGL-1 to AEGL-3, the concentrations increase based on the dose predicted to produce the respective effects (discomfort/irritation vs. disability vs. death). Second, as one moves from shorter exposures (10 min) to longer (8 h), the overall concentration allowed decreases due to the effects of cumulative dose. However, this is not the case for AEGL-2 and AEGL_3 values at 4 h and 8 h, since they represent a threshold for disabling and death outcomes and sometimes only depend on air concentration.

The AEGL values for VC are presented in Table S5 (https://www.epa.gov/aegl/vinyl-chloride-results-aegl-programs, accessed on 3 December 2024). The VC AEGL-1 values were based on mild headaches observed in volunteers. AEGL-2 values are based on effects on the central nervous system, which could impair the ability to escape. Data on cardiac sensitization are supported by lethality data, and are used for AEGL-3 derivation. Thus, at the air exposure equivalent to each AEGL level, we can expect the discomfort/irritation vs. disability vs. death that fit definitions of the AEGL health effects. If biomarkers of VC exposure, such as blood or exhaled breath concentrations, are available, these can be used to link exposure to risk. If exposure biomarkers are unavailable, the PBPK model can be used as a screening tool to predict internal doses after the incident that can result in an adverse AEGL effect.

For example, using reverse dosimetry, Table 4 presents the estimated ranges of peak VC blood levels of VC derived from VC AEGL values. We assume that these peak blood levels can vary by a factor of 3.16 (the square root of 10) among individuals. The assessment factor 3.16 accounts for interindividual variability in toxicokinetics [27].

Following the train derailment, air samples were collected from the VOCs both outside and inside the evacuation area in the days that followed the incident. We converted these samples to determine the vinyl chloride levels, which were then input into Berkeley Madonna to calculate the corresponding arterial blood concentrations. (VC = vinyl chloride; mg/L = milligrams per liter).

When arterial blood concentrations (CA) are determined through biomonitoring, these values can be used to assess whether the at-risk population has likely been exposed to levels exceeding AEGLs. (CA = arterial blood; CX = exhaled breath; CV = venous blood).

## 4. Discussion

This study aimed to integrate data from NTSIP and the exposure investigation to explore the use of PBPK models in emergency risk assessment following exposure to VC. PBPK models are valuable computational modeling approaches in risk assessment because they account for the physiological ADME properties of chemicals in the body [37]. They can also play an important role in an acute chemical incident in determining the predicted value of biomonitoring measurements, assessing the relevance of a biomonitoring study following an acute incident, and identifying the optimal sampling times. For VOCs, typically only about one percent of the exposure biomarker can be measured within several hours after the acute chemical incident.

When complete acute chemical exposure investigations include biomarker measurements—such as the amount of chemical released and its duration—these data can be integrated with PBPK models to predict the concentrations of chemicals in organs/tissues that may pose health risks. Consequently, PBPK modeling could serve as an important tool in acute chemical incidents, offering a quantitative framework to understand and forecast the fate of environmental chemicals in the body following acute chemical exposure. For chemical risk assessment, PBPK models are important for evaluating substances’ pharmacokinetic/toxicokinetic profiles [38].

In this chemical release incident, applying the PBPK model was beneficial in estimating the expected concentration levels and the increments due to the acute exposure. These model estimates may be helpful for emergency risk assessment, providing an initial screening of the VC tissue concentrations. However, evaluating the simulated blood concentration data within a community and among health responders may be challenging due to the lack of available data on blood or urinary levels and the variability of exposure conditions.

VOCs including VC are particularly challenging chemical for PBPK modeling due to several specific limitations. VC can distribute unevenly across different tissues in the body. Modeling this distribution accurately is challenging due to the lack of comprehensive data on tissue-specific partition coefficients and binding affinities. Metabolism of VC and other VOCs is complex, and modeling these metabolic processes requires detailed knowledge of metabolic pathways and rates, which can be difficult to experimentally test, and the data are incomplete or not available yet. Different exposure scenarios can lead to different absorption and distribution patterns during a chemical incident. PBPK models need to account for these variations, which can be challenging given the variability in exposure conditions, variability between individuals and species, and uncertainty in the model parameters. Data gaps exist for VOCs and VC, such as long-term exposure effects and interactions with other chemicals. These gaps can also limit the accuracy and reliability of PBPK models.

PBPK model prediction performance for tissue concentrations is an active research issue. While PBPK models are generally effective, their ability to estimate tissue concentrations varies, particularly between physiological stages (children, pregnancy, elderly, adults, and populations modeling). This emphasizes the importance of continuously validating and refining PBPK models to ensure reliability as data becomes available. The lack of biomarkers for VOC exposure or appropriate biomarkers is also challenging. Identifying reliable biomarkers for VOC exposure is critical for accurately assessing and managing health hazards during chemical incidents. Current research explores noninvasive ways to detect VOCs, such as breath, sweat, and saliva analysis. Nonetheless, these approaches have their limitations.

Due to limited air sampling data, our study focused solely on the simulations for inhalation exposure. This analysis represents a preliminary step that could be expanded to include further analysis, such as inter-individual variability, route extrapolations, other exposure routes, chemical metabolism, and combined multi-route exposure (inhalation, oral, dermal). Our ATSDR human PBPK model toolkit is designed to predict multiple routes of exposure.

Our human VC PBPK model can also be adapted for other VOCs or similar chemicals by substituting the appropriate kinetic parameters. While it provides a useful initial screening, there may be uncertainties due to a lack of chemical-specific kinetic processes (e.g., detailed metabolism pathways equations). A more mechanistic PBPK model could be employed for more detailed applications or fit-for-purpose cases. Different PK/PD/PBPK model structures are tailored for specific purposes. In a chemical incident, a step-by-step approach could be used: identify the chemicals involved, determine the quantity released and its duration, collect samples, and then input these data into the model. The choice between using our generic model structure or a more detailed approach will depend on the specific outcomes needed for the risk assessment.

### Strengths and Limitations

ATSDR PBPK toolkit for high-priority chemicals encompasses many chemicals, from heavy metals to dioxins and volatile organic compounds (VOCs). ATSDR has been recoding highly advanced and efficient PBPK models, translating them onto a single platform (Berkeley Madonna), and modifying them to make them more generic. The resulting recoded models provide increased accessibility to public health assessors and can be more readily applied to the general population. The fidelity of each PBPK model in the toolkit model has also been verified and used by scientists in academia and research institutions [26,39,40]. This PBPK application is important to other ATSDR exposure issues as VC ranks fourth on the agency’s substance priority list [41]. One potential application is to evaluate episodic indoor exposures from vapor intrusion, which often occurs following large rain events. One of the most hazardous exposure situations has involved VC and other chlorinated VOCs [42]. VC and similar chlorinated VOCs made up 7 of the 14 substances.

VOCs including VC are particularly challenging chemicals for PBPK modeling due to several specific limitations. VC can distribute unevenly across different tissues in the body. Modeling this distribution accurately is challenging due to the lack of comprehensive data on tissue-specific partition coefficients and binding affinities. Metabolism of VC and other VOCs is complex, and modeling these metabolic processes requires detailed knowledge of metabolic pathways and rates, which can be challenging to test experimentally, and the data are incomplete or unavailable. Different exposure scenarios can lead to different absorption and distribution patterns during a chemical incident. PBPK models need to account for these variations, which can be challenging given the variability in exposure conditions, variability between individuals and species, and uncertainty in the model parameters. Data gaps exist for VOCs and VC, such as long-term exposure effects and interactions with other chemicals. These gaps can also limit the accuracy and reliability of PBPK models.

PBPK model prediction performance for tissue concentrations is an active research issue. While PBPK models are generally effective, their ability to estimate tissue concentrations varies, particularly between physiological stages (children, pregnancy, elderly, adults, and populations modeling). This emphasizes the importance of continuously validating and refining PBPK models to ensure reliability as data becomes available. The lack of biomarkers for VOC exposure or appropriate biomarkers is also challenging. Identifying reliable biomarkers for VOC exposure is critical for accurately assessing and managing health hazards during chemical incidents. Current research explores noninvasive ways to detect VOCs, such as breath, sweat, and saliva analysis. Nonetheless, these approaches have their limitations.

Our analysis utilized a variety of data sources to gather information on incidents, and the results should be interpreted with caution due to the possibility of under-reporting. One limitation of PBPK modeling in assessing exposure following acute chemical incidents is that only a limited number of chemicals have established PBPK models. A potential next step could involve assembling a task force to develop models for chemicals that are frequently reported in acute incidents in the United States. Additionally, incorporating age and sex factors into PBPK models [43,44]. This is crucial, as many chemicals involved in NTSIP incidents lack health guidance for variable exposure durations, such as those provided by AEGLs.

It is important to note that this study focused solely on acute chemical incidents. Existing research indicates that chronic exposure to VC can lead to severe health issues, and this aspect should be considered for future studies [11,28,37].

Another potential limitation is the availability and accessibility of the Berkeley Madonna software. It may be beneficial to explore alternative tools that can achieve similar outcomes as those examined in this analysis [40,45]. We have provided the code for the model, allowing future users to implement or adapt it as needed.

## 5. Conclusions

In summary, utilizing the PBPK models to assess events such as VC releases can reveal the most and least effective strategies for managing similar incidents. A better understanding of the chemicals involved is essential for preventing future occurrences and ensuring the safety of nearby communities.

We have shown that the PBPK model could serve as a valuable screening tool in acute chemical incidents. PBPK modeling tools could be translated to other acute exposure incidents investigated by ATSDR.

PBPK models can predict the concentration of a chemical in the environment and its subsequent absorption into the body by inhalation or oral or dermal route of exposure. The chemical concentration in the breath, which represents the quantity taken into the bloodstream over a brief period of time, can be estimated using these models. Additionally, PBPK models can predict the chemical’s tissue concentration over an extended period of time, offering information on how the chemical is distributed and excreted from the body. By combining these many elements, PBPK models aid in determining safety guidelines and evaluating the possible health risks connected to acute chemical exposures. The human PBPK toolkit developed at ATSDR offers significant advantages, as risk and health assessment practitioners can readily apply it in the field. Fostering collaboration between model developers and users will promote using and accepting computational tools such as the PBPK model to support the decision-making process. These interactions will enhance the application of such computational modeling approaches in real-world scenarios, increase awareness of their benefits and limitations, and encourage their integration into risk assessment for acute chemical incidents.

## Figures and Tables

**Table 1:** Classification and distribution of chemical categories.

Chemical Category	Examples	Frequency	%
* Volatile organic compounds (VOCs)	Propane, benzene, ethylene, vinyl chloride	3074	19.9
**Other inorganic substances**	Mercury, lithium, sulfur dioxide, hydrogen peroxide	1905	12.3
**Other**	Methamphetamine, diesel fuel, asbestos, carbon dioxide	1836	11.9
**Acids**	Hydrochloric acid, sulfuric acid, phosphoric acid, nitric acid	1765	11.4
**Hydrocarbons**	Butane, oil, natural gas	1373	8.9
**Bases**	Amines, calcium oxide, alkaline hydroxide	1241	8
**Carbon monoxide**	Carbon monoxide	720	4.7
**Ammonia**	Ammonia, ammonium peroxide	670	4.3
**Agriculture chemicals, pesticides**	Ethylene oxide, methylene chloride, nitrobenzene, urea	659	4.3
**Oxy-organics**	Ethylene glycol, propylene glycol, phenol, citric acid	501	3.2
**Mixture across the chemical category**	Mixtures	482	3.1
**Chlorine**	Chlorine, bleach, sodium hypochlorite	456	2.9
**Polymers**	Teflon, polyethylene, fiberglass resin, polypropylene	202	1.3
**Paints and dyes**	Paint, ink	175	1.1
**Hetero-organics**	Aniline, acrylamide	149	1
**Unable to determine**	N/A	95	0.6
**PCBs**	Polychlorinated Biphenyls (PCBs), congeners	62	0.4
**Formulations**	Peroxyacetic acid, concrete admixtures, bioxide	59	0.4
**Missing substance category**	N/A	45	0.3

* VOCs accounted for the most involved in incidents.

**Table 2:** Number of events by year, frequency of chemicals involved, and summary of events.

Year	Frequency			%	
**2010**	2978			22	
**2011**	3128			23.1	
**2012**	3139			23.2	
**2013**	3131			23.1	
**2014**	1153			8.5	
**Number of chemicals involved**	**Frequency**			**%**	
**One**	12,679			82	
**Two**	870			5.6	
**>2**	1920			12.4	
**Release Types**	**Event Type**	**# of Events**	**# of Events w/Injuries**	**# of Injuries**	**# of Fatalities**
**Spill (liquid or solid)**	F	3283	357	791	2
	T	4104	161	257	23
**Volatilization/aerosolized (vapor)**	F	4136	837	2671	69
	T	420	46	95	17
**Fire**	F	120	40	103	1
	T	14	5	10	1
**Explosion**	F	101	63	126	6
	T	7	5	9	0
**Radiation**	F	3		0	0
**Multiple released**	F	1034	309	940	49
	T	255	43	109	20
**Not applicable, threatened release, release type unknown**	T	18	3	4	2
	F	34	5	19	0

F = fixed facility and T = transportation-related incidents; “w/injuries” indicates events that involved reported injuries.

**Table 3:** Predicted arterial blood concentrations using the VC PBPK model after 24 h of exposure outside and inside the evacuation area.

	Vinyl Chloride (ppm)	Arterial Blood Concentration (mg/L)	Vinyl Chloride (ppm)	Arterial Blood Concentration (mg/L)
Date	Outside the Evacuation Area		Inside the Evacuation Area	
**December 1**	0.19	0.0005	5.7	0.014
**December 2**	3.04	0.008	13.11	0.033
**December 3**	57	0.142	39.71	0.1
**December 4**	12.92	0.032	1649.2	4.7
**December 5**	1.33	0.003	0.19	0.0005
**December 6**	0.19	0.0005	1.4	0.003

**Table 4:** Predicted maximum blood and exhaled breath levels by VC PBPK model using AEGLs values.

	AEGL1				AEGL2				AEGL3			
**Time**	**Air Levels (ppm)**	**CA (mg/L)**	**CV (mg/L)**	**CX (mg/L)**	**Air Levels (ppm)**	**CA (mg/L)**	**CV (mg/L)**	**CX (mg/L)**	**Air Levels (ppm)**	**CA (mg/L)**	**CV (mg/L)**	**CX (mg/L)**
**10 min**	450	0.99	0.57	0.86	2800	6.81	4.93	5.87	12,000	29.68	22.28	25.59
**30 min**	310	0.72	0.47	0.62	1600	4.11	3.32	3.55	6800	18.04	15.37	15.55
**60 min**	250	0.6	0.42	0.52	1200	3.18	2.70	2.74	4800	13.23	11.98	11.41
**4 h**	140	0.11	0.25	0.09	820	0.82	1.85	0.71	3400	3.97	8.95	3.42
**8 h**	70	0.17	0.13	0.15	820	2.19	1.89	1.89	3400	9.65	9.11	8.32

## Data Availability

The original contributions presented in this study are included in the article/Supplementary Materials. Further inquiries can be directed to the corresponding author.

## Supplementary Materials

The following supporting information can be downloaded at: https://www.mdpi.com/article/10.3390/jox15020042/s1. Table S1. Open-source tools for air modeling; Table S2. Peak VOC and corresponding VC concentrations within the first hours of the derailment; Table S3. Maximum VOC and corresponding VC concentrations inside the evacuation area; Table S4. Maximum VOC and corresponding VC concentrations outside the evacuation area; Table S5. Acute Exposure Guideline Level for VC: AEGL exposure over time, and the model code.
