# Supplementary material for: Assessing the Application of Physiologically Based Pharmacokinetic Models in Acute Chemical Incidents
Source: J Xenobiot. 2025 Mar 11;15(2):42. doi: 10.3390/jox15020042 (PMC11932312; doi:10.3390/jox15020042)
Supplement: Supplementary file 1 [file jox-15-00042-s001.zip › jox-3379812-supplementary.pdf]

Journal of *Xenobiotics*

MDPI

Supplementary Materials

Assessing the Application of Physiologically Based Pharmacokinetic Models in Acute Chemical Incidents

Sydney Boone [1], Wenjie Sun [2], Pavani Gonnabathula [1], Jennifer Wu [3], Maureen F. Orr [3], M. Moiz Mumtaz [4] and Patricia Ruiz [2,*]

Acronyms/Abbreviations

Acronyms	Meaning
**ACE**	Assessment of Chemical Exposure
**AEGL**	Acute Exposure Guideline Level
**ATSDR**	Agency for Toxic Substances and Disease Registry
**AUC**	The area under the curve
**AUCr**	A ratio for the fit of the VOC (AUC value for the published model or our generic VOC model divided by the AUC value computed from the kinetic data)
**CA**	Arterial blood concentration
**CV**	Venous blood concentration
**CX**	Exhaled breath concentration
**HSEES**	Hazardous Substances Emergency Events Surveillance
**MSSD**	Mean of the sum of the square differences.
**NAICS**	North American Industry Classification System
**NTSIP**	National Toxic Substance Incidents Program
**PBPK**	Physiologically Based Pharmacokinetic
**VC**	Vinyl chloride
**VOC**	Volatile organic compounds

Table S1. Open-source tools for air modeling

Serial No.	Tool Name	Description	Application	Limitation	Reference Link
1	AER-MOD	Versatile, state-of-the-science dispersion model	Regulatory air quality assessments, impact assessments of new sources, environmental impact studies	Requires detailed input data, may not be suitable for complex terrain or complex meteorology	https://www.epa.gov/scram/air-quality-dispersion-modeling-preferred-and-recommended-models
2	AERSCREEN	Screening model for point, area, and line sources	Preliminary air quality assessments, screening of potential sources, and quick evaluation of source impacts	Less accurate than refined dispersion models may not be suitable for complex scenarios	https://www.epa.gov/scram/air-quality-dispersion-modeling-preferred-and-recommended-models
3	CAL-PUFF	Dispersion model for simulating transport and dispersion of air pollutants over large areas	Long-range transport studies, complex terrain modeling, simulations involving multiple sources	Requires detailed input data, computationally intensive	https://19january2017snapshot.epa.gov/scram/air-quality-dispersion-modeling-preferred-and-recommended-models
4	Open-FOAM	Free, open-source CFD software package for simulating fluid flow problems	Research and development of new air dispersion model simulations involving complex geometries or flow conditions	Requires expertise in computational fluid dynamics and programming	https://www.openfoam.com/
5	R	Free, open-source programming language for statistical and graphical analyses	Data analysis, statistical modeling, visualization of air quality data	Limited to data analysis and visualization, not suitable for dispersion modeling	https://www.r-project.org/about.html
6	DAPPLE	Free, open-source air dispersion model for simulating dispersion of pollutants from point, area, and line sources	Long-range transport studies, simulations involving multiple sources, research and development	Requires expertise in air dispersion modeling and programming	https://cgm.engr.ucdavis.edu/facility/1-m-mpr/
7	EPD-AER-MOD	Free, open-source air dispersion model for estimating concentrations of air pollutants from industrial and commercial sources	Air quality modeling in Hong Kong, regulatory assessments of industrial and commercial sources	Limited to Hong Kong, it may not be suitable for other regions	https://www.epd.gov.hk/epd/english/top.html
8	EPA-CMAS	Chemical mechanism and modeling system for simulating the formation and transport of air pollutants	Research and development of air quality models, studying air pollution formation and transport, simulating secondary pollutant formation	Requires expertise in air quality modeling and chemical kinetics	https://www.epa.gov/aboutepa/about-atmospheric-and-environmental-systems-modeling-division

Table S2. Peak VOC and corresponding VC concentrations within the first hours of the derailment.

Area	Time	Peak VOC Value (ppm)	VC Value (ppm)
**N Commerce**	8:33 am	631	1198.9
	8:37 am	760	1444
	8:34 am	694	1318.6
**N Delaware (outside evacuation area)**	8:48 am	108	205.2
	8:44 am	193	366.7
	9:16 am	234	444.6
	8:48 am	111	210.9
	9:48 am	108	205.2
	10:48 am	115	218.5

Since VC levels are 1.9 times higher than the VOC concentration, we found that the peak VC levels were 1444 ppm at North Commerce and 444.6 ppm at North Delaware, respectively (VOC = volatile organic compounds; VC = vinyl chloride; ppm = parts per million)

**Inside Evacuation Area**		
**Date**	Max VOC (ppm)	VC Concentration (ppm)
**November 30, 2012**	4.5	8.55
**December 1, 2012**	3	5.7
**December 2, 2012**	6.9	13.11
**December 3, 2012**	20.9	39.71
**December 4, 2012**	868	1649.2
**December 5, 2012**	0.1	0.19
**December 6, 2012**	0.6	1.14

Table S3. Maximum VOC and corresponding VC concentrations inside the evacuation area

On December 4th, peak VOC levels within the evacuation area reached 868 ppm, with a corresponding VC value of 1649.2 ppm (VOC = volatile organic compounds; VC = vinyl chloride; ppm = parts per million)

Table S4. Maximum VOC and corresponding VC concentrations outside the evacuation area

**Outside Evacuation Area**

Date	Max VOC (ppm)	VC Concentration (ppm)
**November 30, 2012**	0.5	0.95
**December 1, 2012**	0.1	0.19
**December 2, 2012**	1.6	3.04
**December 3, 2012**	30	57
**December 4, 2012**	6.8	12.92
**December 5, 2012**	0.7	1.33
**December 6, 2012**	0.1	0.19

On December 3rd, VOC levels peaked at 30 ppm, with a corresponding VC value of 57 ppm outside the evacuation area . (VOC = volatile organic compounds; VC = vinyl chloride; ppm = parts per million)

Vinyl chloride release from a train derailment

During a train derailment in New Jersey, a punctured tanker car released approximately 24,000 gallons of vinyl chloride near a small town. A shelter-in-place order was initially issued for the surrounding areas, which was repeatedly lifted and reestablished over four days due to fluctuating vinyl chloride levels in the air, influenced by weather conditions. The ACE team, in collaboration with the state and local health departments, undertook the following actions:

- Conducted surveys of community members potentially exposed to VC, hospital staff treating affected patients, and personnel from a facility whose access road was blocked by the derailed train. They also performed hospital chart abstractions. Additionally, state partners mailed a survey to all households in the community.
- Collaborated with a NIOSH team, which interviewed representatives from responder groups and developed a written survey for responders. A report on the NIOSH investigation has been published.
- Addressed responders' questions during their meetings and gathered information to tackle community concerns.
- Reports from investigations of the incident can be accessed from the following links:
    - Paulsboro Fact Sheet on Health Survey Findings/Air Quality Impacts Following Vinyl Chloride Gas Release: September 2014
    - Paulsboro Health Consultation Survey of Residents Following Vinyl Chloride Gas Release: September 2014
    - Paulsboro Air Quality Health Consultation Following Vinyl Chloride Gas Release: September 2014
    - Q & A on Health Effects of Vinyl Chloride: December 2012
    - Q & A on Community and First Responder Health Surveys: December 2012

Additionally, published results of the investigation include:

- Assessment of emergency responders after a vinyl chloride release from a train derailment in New Jersey 2012
- Exposures and symptoms among workers after an offsite train derailment and vinyl chloride release
- Medical Response to a Vinyl Chloride Release From a Train Derailment New Jersey 2012

Table S5. Acute Exposure Guideline Level for VC: AEGL exposure over time

Vinyl Chloride					
	10 minutes	30 minutes	60 minutes	4 hours	8 hours
	ppm				
**AEGL 1**	450	310	250	140	70
**AEGL 2**	2,800	1,600	1,200	820	820
**AEGL 3**	12,000*	6,800*	4,800*	3,400	3,400

AEGL = Acute Exposure Guideline Level

Lower Explosion Limit (LEL) ranges from 38,000 ppm to 293,000 ppm. * = > 10% LEL

https://www.epa.gov/aegl/vinyl-chloride-results-aegl-programs

For values denoted as * safety considerations against hazard(s) of explosion(s) must be considered.

Berkeley Madonna code for PBPK model of Vinyl Chloride

```
;Vinyl Chloride Model

;ATSDR Simulation science section: Mumtaz, M.M.; Ray, M.; Crowell, S.R.; Keys, D.; Fisher, J.; Ruiz, P. Translational Research to Develop a Human Pbpk Models Tool Kit-Volatile Organic Compounds (Vocs). J Toxicol Env Heal A 2012, 75, 6-24, doi:10.1080/15287394.2012.625546.

; Code for oral ingestion of water, inhalation, and dermal transfer of Vinyl Chloride in water

; Physiological parameters can be found in Brown et al., 1997


METHOD RK4

STARTTIME = 0
STOPTIME=24
DT = 1e-4
dtout=.01

BW=70.0          ;Body Weight- kg, Clewell et al., 2000
QPC=24           ;Alveolar ventilation rate- L/H, T. R. Covington et al., 2007
QCC=16.5         ;Cardiac Output- L/H, Clewell et al., 2000
VFC=.214         ;Fraction fat tissue- kg/kg BW, Clewell et al., 2000
VLC=.026         ;Fraction liver tissue- kg/kg BW, Clewell et al., 2000
VBloodC=.079          ;Fraction venous blood- kg/kg BW
VRC=.09          ;Fraction rapidly perfused tissue- kg/kg BW
VSC=.82          ;Fraction slowly perfused tissue- kg/kg BW
VSkC=.051        ;Fraction skin tissue- kg/kg BW
VKC=.044         ;Fraction kidney tissue- kg/kg BW
QFC=.052         ;Fractional blood flow to fat- (L/H)/QC, Clewell et al., 2000
QLC=.24          ;Fractional blood flow to liver- (L/H)/QC, Fisher Mahle and Abbas, 1998
QRC=0.7          ;Fractional blood flow to rapidly perfused- (L/H)/QC, Clewell, 2005
QSC=.30          ;Fract bld flow to slowly perf-(L/H)/QC, Clewell, 2005
QKC=.197         ;Fractional blood flow to kidney- (L/H)/QC
QSkC=.05         ;Fractional blood flow to skin
```

```
SA=19975                                  ;surface area, body-head-cm^2,


{Chemical-Specific Parameters for VC}

PB=1.16                                   ;Blood/air partition coefficient, Clewell, 2001
PL=1.45                                   ;Liver/blood paritition coefficient, Clewell, 2001
PF=20.7                                   ;Fat/blood partition coefficient, Clewell, 2001
PR=1.45                                   ;Rapidly perfused/blood partition coefficient, Clewell, 2001
PS=.83                                            ;Slowly perfused/blood partition coefficient, Clewell, 2001
PK=1.45                                   ;Kidney/blood partition coefficient, (see liver)
PSk=1.45                                  ;Skin/blood partition coefficient, Poet et al., 2000
MW=62.5                                   ;Molecular Weight, g/mole, EPA 2000
Vmaxc=3.97                                ;Maximum velocity of metabolism- mg/h, Reitz, 1996
Km=.04                                            ;Michaelis Menten- mg/L, Reitz, 1996

{Calculated Parameters}

VS=VSC*BW-VF-VSk                                  ;Volume slowly perfused tissue- L
VF=VFC*BW                                         ;Volume fat tissue- L
VK=VKC*BW                                         ;Volume kidney- L
VL=VLC*BW                                         ;Volume liver- L
VBlood=VBloodC*BW                                 ;Volume venous blood- L
VSk=VSkC*BW                                       ;Volume skin- L
VR=VRC*BW-VL-VK                                   ;Volume rapidly perfused tisse- L
QC=QCC*BW**.75                            ;Cardiac output- L/hr
QP=QPC*BW**.75                            ;Alveolar ventilation- L/hr
QF=QFC*QC                                         ;Blood flow to fat- L/h
QL=QLC*QC                                         ;Blood flow to liver- L/h
QR=QRC*QC-QL-QK                                   ;Blood flow to rapidly perfused tissue- L/h
QS=QSC*QC-QF-QSk                                  ;Blood flow to slowly perfused tissue- L/h
QK=QKC*QC                                         ;Blood flow to kidney- L/h
QSk=QSkC*QC                                       ;Blood flow to skin- L/h
VMAX=Vmaxc*BW**.75                                ;max metabolism, mg/hr

{Mass Balance for blood flows and volumes tissues}
Qtot=QS+QR+QSk+QL+QK+QF                   ;sum of blood flows to compartments
Vtot=vs+vr+vk+vf+vsk+vl                   ;sum of tissue volumes
Qbal=Qtot-QC                                      ;check on blood
Vbal=BW-Vtot                                      ;check on volume

{Exposure Parameters}

CONC=0                                    ;Inhaled concentration- ppm, default set=0
inhale_time=5                                     ;Length for inhalation exposure- h
inhale_interval=100000                            ;Period for repeated inh exposures, Set to large value for no repeat dosing
CIX=CONC*MW/24450                                 ;Inhaled concentration- mg/L

;Oral
pdose=0                                   ;oral dose mg/kg/day, default=0
dose_per_drink=(pdose*BW)/number_drink            ;oral dose mg per drink equally divides for each drink
doser=dose_per_drink/drink_time                   ;drink rate/ hr
drink_interval=6                                  ;time between dirnks, set to stoptime for single drink
daily_drink_interval=IF drink_interval>24 THEN 24 ELSE drink_interval      ;sets max daily_drink_interval at 24 for single dirnk
number_drink=24/daily_drink_interval                          ;number of drinks per day
drink_time=.25                                    ;length of time drinking water/ hr

;Dermal
Kp=.015                                           ;Skin permeability constant- cm/hr, Poet et al., 2000
```

```
Cliq=0                                   ;concentration of VC in water-mg/L, Dr. Fisher, default=0
PSkliq=     53                           ;Skin/water partition coefficient, Poet et al., 2000
skin_time=0                         ;Length for Dermal Exp - hr SET =to 0 when no dermal exposure
skin_interval=100000                     ;Period for repeated derm exposures Set to large value for default no repeat dos-
ing

{Exposure Routes}

;Dosing Schedule
;INHALATION
CI=CIX*AIR                                    ;Turning on multiday exposure
AIR=IF MOD(TIME,inhale_interval)>=inhale_time THEN 0 ELSE 1       ;repeated square wave function

;ORAL repeated exposure
ORALH20=IF MOD(TIME,drink_interval)>=drink_time THEN 0 ELSE 1        ;repeated exposure to Vinyl Chloride by oral exposure

;DERMAL exposure in water
SKINH20=IF MOD(TIME,skin_interval)>=skin_time THEN 0 ELSE 1    ;repeated exposure of skin to Vinyl Chloride in water

{Model Equations}

;Chemical in Blood
CA=(QC*CV+QP*CI)/(QC+(QP/PB))                  ;Arterial- mg/L
AVBlood'=(QF*CVF+QL*CVL+QS*CVS+QR*CVR+QK*CVK+QSk*CVSk)-(QC*CV)  ;rate of change in venous blood amount- mg/h
init AVBlood=0                           ;initial amount in venous blood
CV=AVBlood/VBlood                             ;venous concentration

;Exhaled Chemical
CX=CA/PB                            ; Alveolar concentration- mg/L
CXppm=(.7*CX+.3*CI)*24450/MW             ;Exhaled breath concentration
AX'=QP*CX                           ;Amount exhaled
init AX=0                           ;initial amount in exhaled breath
AINH'=QP*CI                              ;Amount inhaled
init AINH=0                         ;initial amount inhaled

;Chemical in Rapidly perfused tissue compartment
AR'=QR*(CA-CVR)                          ;rate of change in viscera amount- mg/h
init AR=0                           ;initial amount in viscera- mg
CR=AR/VR                            ;viscera concentration- mg/L
CVR=CR/PR                           ;concentration in visceral capillary blood- mg/L

;Chemical in Slowly perfused tissue compartment
AS'=QS*(CA-CVS)                     ;rate of change in slowly perfused amount- mg/h
init AS=0                           ;initial amount in slowly perfused tissue- mg
CS=AS/VS                            ;slowly perfused tissue concentration- mg/L
CVS=CS/PS                           ;concentration in SP tissue capillary blood- mg/L

;Chemical in Fat compartment
AF'=QF*(CA-CVF)                     ;rate of change in fat amount- mg/h
init AF=0                           ;initial amount in fat- mg
CF=AF/VF                            ;fat concentration - mg/L
CVF=CF/PF                           ;concentration in fat capillary blood- mg/L

;Chemical in Liver compartment
AL'=QL*(CA-CVL)-RAM+Roral                ;rate of change in liver amount with oral exposure- mg/h
init AL=0                           ;initial amount in liver -mg
CL=AL/VL                            ;liver concentration- mg/L
CVL=CL/PL                           ;concentration in liver capillary blood- mg/L
```

```
;Oral ingestion into the Liver
Roral=doser*ORALH20                          ;repeated oral intake- mg/hr
AORAL'=Roral                                 ;amount ingested-mg
init AORAL=0                                 ;initial amount

;Chemical in Kidney compartment
AK'=QK*(CA-CVK)                              ;rate of change in the kidney amount-mg/h
init AK=0                            ;initial amount in kidney- mg/h
CK=AK/VK                             ;kidney concentration- mg/h
CVK=CK/PK                                    ;concentration in kidney capillary blood- mg/h

;Chemical in Skin compartment
ASk'=QSk*(CA-CVSk)+RASkin                    ;rate of change in skin amount- mg/h
init ASk=0                           ;initial amount in skin- mg/h
CSk=ASk/VSk                                  ;skin concentration- mg/h
CVSk=CSk/PSk                                 ;concentration in skin capillary blood- mg/h

;Chemical across skin
RASkin=(Kp*SA/1000)*(Cliq-CSk/PSkliq)*SKINH20          ;rate of transfer of chemical across skin (flux)-mg/hr with repeated expo-
sure
ASkin'=RASkin                                ;amount of chemical transferred-mg
init ASkin=0                         ;initial amount in skin-mg
ASkinOut'=(Kp*SA/1000)*(CSk/PSkliq)*SKINH20  ;rate of loss through skin (mg/hr)
init ASkinOut=0;                             ;total amount lost through skin (mg)

;Chemical metabolism
RAM=Vmax*CVL/(Km+CVL)                        ;rate of metabolism- mg/h
AM'=RAM                              ;amount metabolized- mg
init AM=0                            ;initial amount metabolized- mg

Daily       = time/24.
DailyAM= AM /(Daily+1e-33)
DailyAMconc=DailyAM/VL

;Mass Balance for inhalation, Check InhDose=Mass
InhDOSE'=QP*(CI-CX)                          ;net absorption- mg/h
init InhDOSE=0                               ;initial net absorption- mg
Mass=AF+AR+AS+AL+AM+AK+ASk+AVBlood                 ;in tissues+metabolized-mg+AVBlood

;Mass Balance for oral intake, Check Oralwater=MASSWATER
Oralwater=Aoral
drink_day=dose_per_drink*number_drink            ;mg ingest each day
MASSWATER=AF+AR+AS+AL+AM+ASk+AK+AX+AVBlood     ;in tissues+metabolized-mg+AVBlood+amount exhaled

;Mass Balance for dermal intake, Check Dermalwater=MASSWater
Dermalwater=ASkin

display cv,mass,QC,cx , Mass, CL, CA, CVL, AM, RAM, DailyAMconc
display BW,QPC,QCC,CONC,AF,AL,AR,AS,AK,ASk,AVBlood,CA, InhDose,Mass,Masswater,Oralwater,Dermalwater,
Cliq,Pdose,skin_time, inhale_time, inhale_interval, skin_interval, drink_interval
```
